# Kidney Disease Management in the Hospital Setting: A Focus on Inappropriate Drug Prescriptions in Older Patients

**DOI:** 10.3389/fphar.2021.749711

**Published:** 2021-10-08

**Authors:** Vincenzo Arcoraci, Maria Antonietta Barbieri, Michelangelo Rottura, Alessandro Nobili, Giuseppe Natoli, Christiano Argano, Giovanni Squadrito, Francesco Squadrito, Salvatore Corrao, on behalf of the REPOSI investigators

**Affiliations:** ^1^ Department of Clinical and Experimental Medicine, University of Messina, Messina, Italy; ^2^ Department of Neuroscience, IRCCS Istituto di Ricerche Farmacologiche Mario Negri, Milan, Italy; ^3^ Dipartimento di Promozione Della Salute, Materno Infantile, Medicina Interna e Specialistica di Eccellenza “G. D’Alessandro”, PROMISE, University of Palermo, Palermo, Italy; ^4^ SunNutraPharma, Academic Spin-Off Company of the University of Messina, Messina, Italy; ^5^ Department of Internal Medicine, National Relevance and High Specialization Hospital Trust ARNAS Civico, Di Cristina, Benfratelli, Palermo, Italy

**Keywords:** chronic kidney disease, appropriateness of prescriptions, prescribing patterns, real-world data, hospital setting, older patients

## Abstract

Aging with multimorbidity and polytherapy are the most significant factors that could led to inappropriate prescribing of contraindicated medications in patients with chronic kidney disease (CKD). The aim of this study was to evaluate the prescriptions of contraindicated drugs in older adults in CKD and to identify their associated factors in a hospital context. An observational retrospective study was carried out considering all patients ≥65 years with at least one serum creatinine value recorded into the REPOSI register into 2010–2016 period. The estimated glomerular filtration rate (eGFR) was applied to identify CKD. A descriptive analysis was performed to compare demographic and clinical characteristics; logistic regression models were used to estimate factors of inappropriate and percentage changes of drug use during hospitalization. A total of 4,713 hospitalized patients were recorded, of which 49.8% had an eGFR <60 ml/min/1.73 m^2^; the 21.9% were in treatment with at least one inappropriate drug at the time of hospital admission with a decrease of 3.0% at discharge (*p* = 0.010). The probability of using at least one contraindicated drug was significantly higher in patients treated with more several drugs (OR 1.21, 95% CI 1.16–1.25, p <0.001) and with CKD end-stages (G4: 16.90, 11.38–25.12, *p* < 0.001; G5: 19.38, 11.51–32.64, *p* < 0.001). Low-dose acetylsalicylic acid was the contraindicated drug mainly used at the time of admission, reducing 1.2% at discharge. An overall increase in therapeutic appropriateness in hospitalized older patients with CKD was observed, despite a small percentage of therapeutic inappropriateness at discharge that underlines the need for a closer collaboration with the pharmacologist to improve the drug management.

## Introduction

Chronic kidney disease (CKD) is considered one of the most serious public health problems in the world (Genovese et al., 2018; Ammirati, 2020). The CKD is classified into stages according to glomerular filtration rate (GFR) and albuminuria values with GFR values < 60 ml/min/1.73 m^2^ indicating a CKD classified from G3a to G5, while GFR values between 60 ml/min/1.73 m^2^ and 89 ml/min/1.73 m^2^ indicate a CKD classified as G2 only if concomitant renal damage has been detected (KDIGO, 2013).

The prevalence of CKD is estimated as 9.1% worldwide, and, in detail, stage G3 accounts for 3.9%, stage G4 for 0.2%, and stage G5 for 0.1% (Bikbov et al., 2020). However, the number of people with CKD is expected to increase worldwide with population growth and aging. Indeed, it is widely known that kidney disease is one of the most comorbid conditions in older adults, with an overall estimated prevalence ranging from 21.4 to 47.0% and with an incidence rate of stage G4 that rises with older age (Matsushita et al., 2010; Amaral et al., 2019; Ravani et al., 2020). Aging with multimorbidity and polytherapy are the most significant factors associated with the onset and progression of end-stages of CKD but also with the hospitalization rate (Mallappallil et al., 2014; Wong et al., 2019; Schrauben et al., 2020). Moreover, the presence of CKD could be associated with several comorbidities, including hypertension, diabetes, chronic respiratory and cardiovascular disorders (Fox et al., 2012; Mahmoodi et al., 2014; Fraser et al., 2015).

Patients with at least one comorbidity are usually treated with a higher number of drugs, excreted mainly by the kidney. Older adults with GFR <60 ml/min/1.73 m^2^ may have altered renal excreted drug pharmacokinetics and an increased risk of drug interactions which usually leads to an adjustment of their dosages to avoid toxicity (KDIGO, 2013). Older people take a broad number of medications daily, thus increasing the probability of inappropriate prescriptions (Formica et al., 2018; Roux-Marson et al., 2020). Moreover, the use of inappropriate drug prescriptions could lead to a possible worsening and progression of CKD (Schmidt et al., 2019). Several drugs, including renin-angiotensin-system (RAS)-blocking agents, anticoagulants, hypoglycemic, beta-blockers, antibiotics, analgesics, lithium, non-steroidal anti-inflammatory drugs (NSAIDs), and some antineoplastic agents are commonly known to be nephrotoxic (Taber and Pasko, 2008; Okoro and Farate, 2019; Pou et al., 2019).

The burden of CKD arouses the interest from the healthcare system for a more careful appraisal of the GFR values into the management of drug prescriptions. A recent Italian retrospective population-based study in a general practice setting showed that 56.8% of CKD affected patients received inappropriate prescriptions with a significantly greater probability when treated with more drugs, with more comorbidities and during the end stages of CKD (Barbieri et al., 2020). Nevertheless, the management of older patients with GFR <60 ml/min/1.73 m^2^ in the hospital setting is a little-discussed issue in recent literature (Tesfaye et al., 2019; Roux-Marson et al., 2020; Troncoso-Mariño et al., 2021). Hospital admissions provide an opportunity to re-evaluate treatment regimens in patients with GFR <60 ml/min/1.73 m^2^ and, better familiarity with nephrotoxic drugs by a physician in collaboration with a pharmacologist, could help identify appropriate preventive strategies for the management of these patients at discharge. For the reasons described above, this study aimed to evaluate the prescriptions of nephrotoxic and contraindicated drugs in older patients with GFR<60 ml/min/1.73 m^2^ and to identify their associated factors in a real-world context.

## Materials and Methods

### Study Design and Data Collection

An observational retrospective study was carried out using the REgistro POliterapie SIMI (REPOSI) database and considering all data collected from January 2010 to December 2016. The REPOSI is a multicenter collaborative observational registry cooperatively established by the Italian Society of Internal Medicine (SIMI), IRCCS Ca’ Granda Maggiore Policlinico Hospital Foundation and Mario Negri Institute of Pharmacological Research IRCCS that was made up to recruit, monitor and evaluate hospitalized older adults ≥65 years admitted to 102 Italian internal medicine and geriatric wards with data came from each single medical record and collected every 2 years from 2008 onwards (Nobili et al., 2011; Argano et al., 2020). The study protocol was approved by the local Ethics Committee of the responsible hospitals, and the creation of a database was conducted according to Good Clinical Practice recommendations and the Declaration of Helsinki while maintaining the anonymity of patients. All diagnoses were coded according to the International Classification of Diseases ninth Edition (ICD-9) system and prescribed drugs at admission and discharge were assessed by the Anatomic Therapeutic Chemical (ATC) Classification System.

**FIGURE 1:**
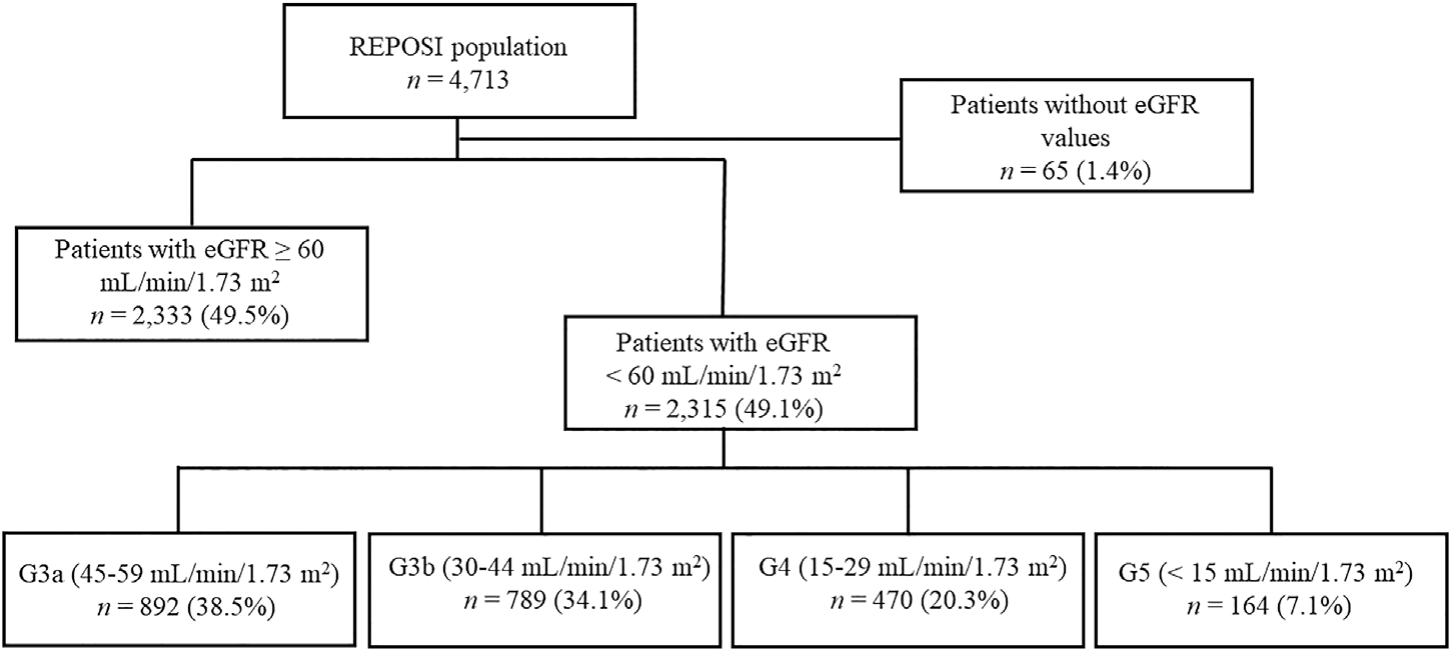
The flow chart describes the patients reported into the REPOSI database according to the eGFR values.

The sample size included all patients ≥65 years and with at least one serum creatinine value registered during the hospitalization and collected into the REPOSI database. The estimated GFR (eGFR) was calculated according to the respective creatinine value for each patient with the CKD-epidemiology collaboration (CKD-EPI) formula, retrospectively; patients with eGFR <60 ml/min/1.73 m^2^ were defined as CKD patients. Moreover, patients were classified into stages G3a—G5 based on the eGFR values: stage G3a (eGFR 45–59 ml/min/1.73 m^2^), G3b (eGFR 30–44 ml/min/1.73 m^2^), G4 (eGFR 15–29 ml/min/1.73 m^2^), and G5 (eGFR <15 ml/min/1.73 m^2^) (Figure 1) (KDIGO, 2013).

Socio-demographic characteristics reported in each medical record and including age, sex, height, weight, body mass index (BMI), information on lifestyles, clinical features such as eGFR values, comorbidity and severity indexes including the Cumulative Illness Rating Scale Severity Index (CIRS.S) and the Cumulative Illness Rating Scale Comorbidity Index (CIRS.C) (Linn et al., 1968; Corrao et al., 2020), performance in basic activities of daily living measured by the Barthel Index (BI) (Mahoney and Barthel, 1965; Corrao et al., 2019), the presence of mood disorders using the Geriatric Depression Scale (GDS) (Hickie and Snowdon, 1987; Argano et al., 2021) were recovered during the index hospitalization. Moreover, length of hospitalization, diagnosis of CKD registered by a physician, comorbidities, and mortality were also recorded. Drugs used up to the admission and prescribed after discharge were considered separately. Patients died during the hospital stay were excluded from the evaluation of drugs prescribed at discharge.

The use of nephrotoxic drugs was evaluated, and, according to the clinical conditions of each patient, the contraindicated prescriptions were identified as inappropriate. Specifically, patients were classified based on the inappropriate drug used in:

• *Fully appropriate,* if they were being treated with non-contraindicated drugs at admission and discharge;
• *Fully inappropriate,* if they had at least one contraindicated drug used at the time of admission and discharge;
• *Appropriate at discharge,* if they were in treatment with contraindicated drugs at admission, but this use was discontinued at discharge;
• *Inappropriate at discharge,* if they have not contraindicated treatment at admission, but they have started at least one contraindicated drug was started at discharge.

### Definition Of Nephrotoxicity

Nephrotoxic drugs were identified through a literature review using the Medical Subject Headings (MeSH) terms “nephrotoxic drug” and “drug-induced renal failure”. Moreover, an assessment of cautionary notes for prescribing in people with eGFR <60 ml/min/1.73 m^2^ was performed (KDIGO, 2013). After a careful consideration, all drugs were categorized as “contraindicated drugs” and therefore inappropriate by checking their Summary of Product Characteristics (SmPC) available at the time of the study (Supplementary Table 1) (Agenzia Italiana del Farmaco, 2021).

### Statistical Analyses

A descriptive analysis was performed to compare all the clinical and demographic characteristics of the study population among patients with eGFR <60 ml/min/1.73 m^2^ and patients with eGFR ≥60 ml/min/1.73 m^2^. The categorical variables and continuous variables were reported as absolute and relative frequencies with 95% confidence intervals (CIs) and as medians with interquartile range (Q1-Q3), respectively. Considering a not normal distribution of some of the numerical variables after the use of the Kolmogorov–Smirnov test for normality, a non-parametric approach was adopted. The Mann–Whitney *U* test for independent sample and two-tailed Pearson chi-squared test were carried out to compare continuous variables and categorical variables, respectively.

In patients affected by a CKD-stage ≥ G3a, univariate logistic regression models were performed to identify the factors associated with contraindicated drug use (sex, age, BMI, CIRS. S, CIRS. C, BI, GDS, registered diagnosis of CKD, year of hospitalization, stage of eGFR values, number of active substances used at admission and discharge, and length of hospital stay), on discharge, using patients without inappropriate prescriptions as comparators.

All variables, identified as factors associated with contraindicated drug use in the univariate models were included in a stepwise multivariate logistic regression model (backward elimination procedure, *α* = 5%). Moreover, all variables not resulted significant in the univariate analysis, but considered clinically remarkable after a careful consideration based on current knowledge and clinical expertise, and with a cut-off of alpha error of 0.2 according to Hosmer–Lemeshow test, were also included (Sun et al., 1996; VanderWeele, 2019). Conversely, variables with the same clinically significant and with a plausible collinearity, verified by the Spearman’s rank correlation coefficient, were excluded from the multivariate model. Odds ratios (ORs) with 95% CIs were calculated for each covariate of interest in univariate (crude OR) and multivariate (adjusted OR) regression models. The goodness of fit of the regression model was carried out by the Hosmer–Lemeshow test for adequacy. Moreover, percentage differences and Δ percentage changes from admission to discharge were executed to assess the use of inappropriate active substances during hospitalization. A *p-value* < 0.05 was considered statistically significant. Statistical analysis was performed using the SPSS version 23.0 (IBM Corp. SPSS Statistics).

## Results

### Characteristics of Patients

During the study period, a total of 4,713 hospitalized patients were recorded into the REPOSI register, of which 4,648 (98.6%) had at least one reported serum creatinine value. An eGFR <60 ml/min/1.73 m^2^ was found in 2,315 (49.8%) patients; nevertheless, a diagnosis of CKD registered by the physician was retrieved only in 37.6% of cases. Taking into account eGFR values, patients were mostly in G3a and G3b stages (*n* = 892; 38.5% and *n* = 789; 34.1%, respectively) followed by subjects in G4 and G5 (*n* = 470; 20.3%; *n* = 164; 7.1%).

A higher prevalence of females and older was observed in subjects with eGFR <60 ml/min/1.73 m^2^ compared to subjects with eGFR ≥60 ml/min/1.73 m^2^ (*p* < 0.001 for both comparisons). Moreover, patients with eGFR <60 ml/min/1.73 m^2^ had a significant higher value of all comorbid indices, a higher length of hospital stay, died during the hospital stay, and were in treatment with a significantly greater number of different drugs per patient at admission (median 5, Q1-Q3 4–8 *vs* 4, 3–6) and discharge (6, 3–9 *vs* 5, 3–8). Hypertension, diabetes mellitus, heart failure, atrial fibrillation, ischemic heart disease, anemia, mood disorders, peripheral vascular disease, hypertensive heart disease, and atherosclerosis were more frequently described in patients with eGFR <60 ml/min/1.73 m^2^ (*p* < 0.05 for all comparisons) (Table 1).

**TABLE 1:** Socio-demographic characteristics of patients with an estimated glomerular filtration rate (eGFR) ≥60 ml/min/1.73 m^2^ *vs* patients with an eGFR <60 ml/min/1.73 m^2^.

Characteristic		eGFR ≥60 ml/min/1.73 m^2^ (*n* = 2,333)	eGFR <60 ml/min/1.73 m^2^ (*n* = 2,315)	*P* Value
**Sex (M), *n* (%)**	1,205 (51.7)		1,069 (46.2)	<0.001
**Age, median (Q1-Q3)**	78 (72–83)		82 (76–86)	<0.001
**BMI, median (Q1-Q3)**	25.2 (22.7–28.0)		25.7 (22.7–29.1)	0.002
**CIRS.C, median (Q1-Q3)**	3 (1–4)		3 (2–5)	<0.001
**CIRS.S, median (Q1-Q3)**	1.54 (1.38–1.77)		1.69 (1.46–1.92)	<0.001
**BI >10, *n* (%)**	677 (32.1)		831 (40.1)	<0.001
**GDS >2, *n* (%)**	342 (17.3)		376 (19.7)	0.048
**Registered CKD diagnosis, *n* (%)**	71 (3.0)		871 (37.6)	<0.001
**Number of active substances at admission, median (Q1–Q3)**	4 (3–6)		5 (4–8)	<0.001
**Number of active substances at discharge, median (Q1–Q3)**	5 (3–8)		6 (3–9)	<0.001
**Length of hospital stay, median (Q1-Q3)**	9 (6–14)		9 (6–14)	0.009
**Comorbidities**, ***n* (%)**				
Anemia	407 (17.4)		537 (23.2)	<0.001
Arthropathies	304 (13.0)		308 (13.3)	0.782
Atrial fibrillation	473 (20.3)		678 (29.3)	<0.001
Cerebrovascular disease	75 (3.2)		78 (3.4)	0.768
COPD	443 (19.0)		480 (20.7)	0.136
Diabetes mellitus	606 (26.0)		762 (32.9)	<0.001
Gastritis and duodenitis	309 (13.2)		303 (13.1)	0.875
Heart failure	322 (13.8)		613 (26.5)	<0.001
Hyperplasia of prostate	275 (11.8)		254 (11.0)	0.381
Hypertension	1,297 (55.6)		1,450 (62.6)	<0.001
Hypertensive heart disease	271 (11.6)		334 (14.4)	0.004
Ischaemic heart disease	444 (19.0)		616 (26.6)	<0.001
Mood disorders	298 (12.8)		375 (16.2)	0.001
Neoplasms	425 (18.2)		400 (17.3)	0.403
Peripheral vascular disease^a^	375 (16.1)		445 (19.2)	0.005
**Number of deaths, n (%)**	74 (3.2)		135 (5.8)	<0.001

*BMI*, Body Mass Index; *CIRS.S*, Cumulative Illness Rating Scale Severity Index; *CIRS.C*, Cumulative Illness Rating Scale Comorbidity Index; *BI*, Barthel Index; *GDS*, Geriatric Depression Scale; *COPD*, Chronic obstructive pulmonary disease.

a Includes all ICD-9 codes from 440 to 459.9.

### Characteristics of Drugs Used

Patients with eGFR <60 ml/min/1.73 m^2^, treated with at least one inappropriate drug at the time of hospital admission were 508 (21.9%) and this data decreased by up to 18.9% at discharge (*p* = 0.010). The reduction of inappropriate drug use during the hospital stay was observed particularly in the end-stage of CKD with a significant percentage reduction in G4 patients (−13.2%; *p* = 0.020) (Table 2).

**TABLE 2:** Inappropriate drug use at admission and discharge in patients with eGFR <60 ml/min/1.73 m^2^.

Stage of CKD, *n* (%)^a^	Admission	Discharge	Percentage reduction	*P* Value
G3a	57 (6.4)	55 (6.4)	0.4	0.981
G3b	75 (9.5)	63 (8.4)	11.6	0.448
G4	275 (58.5)	220 (50.8)	13.2	0.020
G5	101 (61.6)	73 (52.1)	15.3	0.097
Total	508 (21.9)	411 (18.9)	14.1	0.010

*CKD*, Chronic kidney disease.

a Percentages were calculated on the total number of patients for each stage of CKD at admission (G3a = 892; G3b = 789; G4 = 470; G5 = 164) and at discharge (G3a = 857; G3b = 750; G4 = 433; G5 = 140).

Most of patients were fully appropriate (*n* = 1,642; 70.9%) and the 5.5% (*n* = 127) were discharged with appropriate prescriptions. Otherwise, the 14.5% of subjects were fully inappropriate (*n* = 335) and patients discharged with inappropriate medications were 76 (3.3%). Patients died during the hospital stay (*n* = 135) were not included in any group. The most inappropriately used drug classes during hospitalization and at discharge were antithrombotic agents (56.4%), diuretics (23.0%), antidiabetics (9.1%), analgesics (4.2%), anti-inflammatory and antirheumatic drugs (3.9%), and beta blockers (3.9%).

The number of patients treated with several inappropriate drugs increased from early to the end stages of CKD both at admission (G3a = 6.4%; G3b = 9.5%; G4 = 58.5%; G5 = 61.6%) and discharge (G3a = 6.4%; G3b = 8.4%; G4 = 50.8%; G5 = 52.1%). The probability of using at least one contraindicated drug in patients with eGFR <60 ml/min/1.73 m^2^ after hospitalization was significantly higher in patients treated with more drugs (Adj OR 1.21 (95% CI 1.16–1.25), *p* < 0.001). The severity of CKD was also an independent factor of inappropriate drug use (G4: 16.90 (11.38–25.12), *p* < 0.001; G5: 19.38 (95% CI 11, 51–32.64), *p* < 0.001). However, gender, age, CIRS. C, CIRS. S, BI, GDS, year of hospitalization, length of hospital stay, and the presence of a registered diagnosis of CKD were not factors associated with contraindicated drug use at discharge (Table 3). No differences were shown at admission or discharge.

**TABLE 3:** Factors associated with contraindicated drug use in patients with an eGFR <60 ml/min/1.73 m^2^ at discharge.

Variable	Crude OR (95% CI)	*P* Value	Adjusted OR^a^ (95% CI)	*P* Value
**Age (years)**	1.02 (1.00–1.03)	0.014	1.01 (0.99–1.03)	0.259
**Sex (M)**	1.13 (0,91–1.40)	0.268	1.20 (0.89–1.62)	0.232
**BMI**	1.03 (1.01–1.05)	0.009	1.01 (0.98–1.04)	0.415
**CIRS.S**	4.17 (3.07–5.66)	<0.001	1.41 (0.91–2.17)	0.125
**CIRS.C**	1.26 (1.19–1.33)	<0.001	0.99 (0.83–1.20)	0.989
**BI >10**	1.31 (1.04–1.65)	0.021	1.17 (0.86–1.60)	0.329
**GDS >2**	0.93 (0.68–1.26)	0.620	—	
**Year of hospitalization**	—	—	—	—
2010	—	—	—	—
2012	0.96 (0.69–1.34)	0.831	—	—
2014	0.87 (0.62–1.21)	0.398	—	—
2016	1.19 (0.86–1.65)	0.296	—	—
**Registered CKD diagnosis**	2.96 (2.37–3.69)	<0.001	0.92 (0.66–1.29)	0.639
**CKD stage**	—	—	—	—
CKD stage G3a^b^	—	—	—	—
CKD stage G3b^b^	1.34 (0.92–1.95)	0.130	1.20 (0.77–1.87)	0.420
CKD stage G4^b^	15.06 (10.81–20.99)	<0.001	16.90 (11.38–25.12)	<0.001
CKD stage G5^b^	15.89 (10.34–24.42)	<0.001	19.38 (11.51–32.64)	<0.001
**Number of drugs**	1.19 (1.16–1.22)	<0.001	1.21 (1.16–1.25)	<0.001
**Length of hospital stay (days)**	1.00 (0.99–1.01)	0.932	0.99 (0.98–1.01)	0.358

*CI*, confidence interval; *BMI*, Body Mass Index; *CIRS.S*, Cumulative Illness Rating Scale Severity Index; *CIRS.C*, Cumulative Illness Rating Scale Comorbidity Index; *BI*, Barthel Index; *GDS*, Geriatric Depression Scale; *CKD*, chronic kidney disease; *eGFR*, estimated glomerular filtration rate; *OR*, Odds Ratio.

a Adjusted for all variables considered in the univariate logistic regression model.

b Range codified by each eGFR category: G3a between 45 and 59 ml/min/1.73 m^2^; G3b between 30 and 44 ml/min/1.73 m^2^; G4 between 29 and 15 ml/min/1.73 m^2^ and G5 <15 ml/min/1.73 m^2^.

Patients with CKD were treated with 42 different contraindicated drugs at admission mainly low-dose acetylsalicylic acid (*n* = 309; 13.3%), canrenone (*n* = 73; 3.2%), and metformin (*n* = 44; 1.9%). The number of inappropriate drugs was reduced to 35 various active substances at discharge; of them, the drugs inappropriately used in most patients were low-dose acetylsalicylic acid (*n* = 265; 12.2%), canrenone (*n* = 70; 3.2%), and spironolactone (*n* = 28; 1.3%). Drugs with the greatest percentage reduction during discharge respect to admission were diclofenac (from 7 patients to 1 patient; −84.8%), glimepiride (from 15 to 3; -78.8%), metformin (from 44 to 17, −59.0%), and olmesartan (from 5 to 2; −57.5%). Nevertheless, only 3 medications had an increase in inappropriate use: fondaparinux (from 4 to 7; +85.8%), calcium carbonate (from 8 to 11; +46.6%), and antacids (from 6 to 8; +41.6%). Concerning Δ% from hospitalization to discharge a greater reduction was observed for low-dose acetylsalicylic acid and metformin (−1.2% and −1.1%, respectively) (Table 4).

**TABLE 4:** Most commonly contraindicated drugs (n ≥ 5) in patients with eGFR <60 ml/min/1.73 m^2^ at admission and discharge.

ATC	Medication	Admission	Discharge	Percentage (%) difference	Δ% Discharge-admission
B01AC06	Acetylsalicylic acid	309 (13.3)	265 (12.2)	−8.9	−1.2
C03DA02	Canrenone	73 (3.2)	70 (3.2)	−1.8	−0.1
A10BA02	Metformin	44 (1.9)	17 (0.8)	−59.0	−1.1
C03DA01	Spironolactone	35 (1.5)	28 (1.3)	−15.0	−0.2
N02BA01	Acetylsalicylic acid	24 (1.0)	20 (0.9)	−11.5	−0.1
C07AB12	Nebivolol	24 (1.0)	18 (0.8)	−20.4	−0.2
C10AA07	Rosuvastatin	23 (1.0)	19 (0.9)	−12.3	−0.1
A10BB12	Glimepiride	15 (0.6)	3 (0.1)	−78.8	−0.5
C08CA13	Lercanidipine	14 (0.6)	9 (0.4)	−31.7	−0.2
G04CA01	Alfuzosin	11 (0.5)	8 (0.4)	−22.8	−0.1
A12AA04	Calcium carbonate	8 (0.3)	11 (0.5)	+46.6	+0.2
B01AB06	Nadroparin	7 (0.3)	5 (0.2)	−24.1	−0.1
M01AB05	Diclofenac	7 (0.3)	1 (0.0)	−84.8	−0.3
A02AD01	Antacids	6 (0.3)	8 (0.4)	+41.6	+0.1
N06AX21	Duloxetine	5 (0.2)	5 (0.2)	+6.2	0.0
C09CA08	Olmesartan	5 (0.2)	2 (0.1)	−57.5	−0.1
B01AX05	Fondaparinux	4 (0.2)	7 (0.3)	+85.8	+0.1

*ATC*, Anatomic Therapeutic Chemical.

## Discussion

This is the first study evaluating factors associated with contraindicated drug use in older adults with CKD in internal medicine and geriatric wards. To the best of our knowledge, few studies concerning inappropriate drug prescription in patients followed by other specialists or by general practitioners are available (Barbieri et al., 2020; Chahine, 2020; Roux-Marson et al., 2020; Troncoso-Mariño et al., 2021).

In this context, older patients with eGFR <60 ml/min/1.73 m^2^ were mainly women: the CKD mostly affects females due to gender differences in renal pathophysiology (Cobo et al., 2016; Carrero et al., 2018). A diagnosis of CKD was registered in only 37.6% of patients even if a recent study found a formally documented diagnosis of CKD into the 59% of hospitalized older patients (Tesfaye et al., 2019). Moreover, patients with eGFR <60 ml/min/1.73 m^2^ were particularly affected by cardiovascular disorders including hypertension, atrial fibrillation and heart failure and metabolic disease as well as diabetes. The common presence of multimorbidity in patients with CKD has been linked to a closer decline in renal function (Lee et al., 2018). Hypertension and diabetes were the most observed comorbidities in several studies (Lee et al., 2018; Chahine, 2020). It is well known that heart failure is considered the primary diagnosis at the time of hospitalization of patients with CKD and it is most related to the end-stage (Hakopian et al., 2019; Tesfaye et al., 2019; Roux-Marson et al., 2020; Schrauben et al., 2020).

The evaluation of drug used in patients with CKD showed that the number of drugs at the time of admission ranged from 4 to 8 different active substances for each patient. Several studies showed that the presence of more comorbidities could lead to polytherapy with many drugs used for each patient (Schmidt et al., 2019). Most patients were fully appropriate, and only 22.0% were in treatment with at least one inappropriate drug at admission. This data are lower than that observed in other studies, with percentages ranging from 34.1 to 90.0% (Chahine, 2020; Molnar et al., 2020; Roux-Marson et al., 2020). Moreover, remodeling of therapies during hospitalization was reported in about the 4.0% of subjects, and a reduction in the inappropriate use of medications at discharge mainly occurred in patients at the end-stage of CKD (G4 and G5). This finding agreed with the results of another study that showed a significant decrease in inappropriate drugs from admission to discharge, highlighting once again the importance of a careful assessment by the clinician (Tesfaye et al., 2019).

The recorded diagnosis of CKD did not modify the percentage of patients treated with at least one contraindicated drug at discharge, as previously noted (Barbieri et al., 2020). Specifically, the increase in the number of different drugs taken and the severity of CKD were the factors associated with an increased risk of inappropriateness. Further evidence showed that the number of several medications was the most factor of inappropriate prescription (Chang et al., 2015; Doody et al., 2015; Laville et al., 2018; Chahine, 2020). The correlation between inappropriateness and the severity of CKD was controversial. In line with other findings (Laville et al., 2018; Barbieri et al., 2020), the CKD stage was an independent factor of contraindicating drug use. Otherwise, no significant difference was noticed in the use of potentially inappropriate medications in patients in advanced CKD stages (Chahine, 2020) and patients at the CKD stage G3 were significantly more likely to receive at least one nephrotoxic drug compared to those at end-stages (Okoro and Farate, 2019).

Concerning the use of every single active substance, low-dose acetylsalicylic acid (13.3% of inappropriate drugs) was the mostly used medication at the time of admission. This consumption was reduced by −1.2% at discharge even if it was considered the first inappropriate drug also at discharge (12.2% of patients). Specifically, acetylsalicylic acid was used in these patients as a platelet aggregation inhibitor. A higher use of acetylsalicylic acid in patients with CKD was observed in other studies (Ingrasciotta et al., 2014; Abd ElHafeez et al., 2019; Okoro and Farate, 2019; Barbieri et al., 2020). Patients mostly treated with low-dose acetylsalicylic acid were those with a registered diagnosis of CKD. This could be justified by the comorbid peripheral vascular disease and ischemic heart conditions and could be explained because no valid therapeutic alternative to low-dose acetylsalicylic acid is available in clinical practice for these certain conditions. Moreover, the most recent KDIGO guidelines on the management of patients with CKD highlight the importance to use aspirin to prevent cardiovascular events and suggest to not stopped this therapy in case of diabetes or nervous and/or cardiovascular diseases, always considering the higher risk of major bleeding due to the defective platelet adhesion and aggregation, and other intrinsic platelet defects in patients with CKD (KDIGO, 2013; Gallagher et al., 2019). Other contraindicated drugs that experienced a reduction in use at discharge were diclofenac, glimepiride, metformin, and olmesartan. Reduction in their consumptions could be related to substitution with other alternative therapeutics, including glucagon-like peptide-1 receptor agonists (GLP-1 RA) and thiazolidinediones (Barone et al., 2017; Kristensen et al., 2019) or analgesics (Baker and Perazella, 2020). The NSAIDs, comprising diclofenac, should be avoided in older CKD subjects even if their consumptions continue to be frequent after discharge because of their availability over the counter (Mirishova and Hammad, 2018). Concerning angiotensin receptor blockers, guidelines recommended their use as first-line therapy in patients with CKD but may cause hyperkalemia (Ku et al., 2019; Sinha and Agarwal, 2019). However, the use of olmesartan is contraindicated when concomitantly administered with aliskiren in patients with CKD for the higher risk of adverse effects (Agenzia Italiana del Farmaco Banca Dati Farmaci, 2021). The use of metformin must always be avoided in patients with advanced CKD stage because it leads to an increased risk of lactic acidosis (Tanner et al., 2019; Molnar et al., 2020). However, the incidence of lactic acidosis associated with its use has been negligible, and guidelines are largely flexible, mainly in the mild-moderate stages of CKD (Lazarus et al., 2018). Moreover, it could be essential to pose attention to the drug-drug interactions, including the concomitant use of low-dose acetylsalicylic acid and metformin that could result into a moderate severity of type C (Santos-Díaz et al., 2020).

Another critical concern was related to the increase in the inappropriate use of fondaparinux, calcium carbonate, and antacids after hospitalization because they often did not have valid alternatives. Fondaparinux is preferred when no other choice is available including warfarin and other anticoagulant drugs (Saheb Sharif-Askari et al., 2017); however, it is associated with a higher probability of thrombocytopenia and bleeding especially in older patients for a reduced drug elimination (Agenzia Italiana del Farmaco, 2021). Calcium carbonate is used in CKD patients due to common hypocalcaemia or as a phosphate binder if alternatives cannot be found; however, in a recent study, it was shown that calcium carbonate was associated with increased vascular calcification that makes calcium-based phosphate binders not a safe option for CKD patients (Neto and Frazão, 2021); the last Clinical Practice Guideline Update for the Diagnosis, Evaluation, Prevention, and Treatment of CKD-Mineral and Bone Disorder suggested that excess exposure to calcium through diet, medications, or dialysate may be harmful across all GFR categories of CKD. Nevertheless, available evidence does not conclusively demonstrate that calcium-free agents are superior to calcium-based agents leaving the choice of possible alternative therapies (Kidney Disease: Improving Global Outcomes, 2017). Overall, the hospital doctor of the internal medicine and geriatrics wards sees quite complex patients at the time of admission, mainly older adults, in polytherapy and with multi-organ damage that could compromise the patient’s own life. For this reason, physicians prioritize the clinical stabilization of the patient for the entire duration of the hospitalization. This could overshadow some more competent evaluations from the point of view of pharmacologist and consequently neglect, at least initially, considerations regarding the pharmacokinetic and pharmacodynamic characteristics of the drugs used during hospitalization and the possible consequences of toxicity on some organs, including nephrotoxicity. At discharge, the patient’s general condition should be improved, and drug therapy may be adjusted according to the new clinical status. In some cases, no valid therapeutic alternatives with better efficacy and safety profiles than those already administered to the patient are available. Moreover, frequent monitoring of renal markers could be useful to constantly re-evaluate the risk/benefit profile of drugs highlighting the concept of personalized drug therapy. Additionally, the clinical pharmacologist involvement, a physician commonly consulted in European Hospitals due to his expertise in drug therapy and drug-drug interactions, becomes really important in the multidisciplinary management of patients with CKD. It improves the appropriate medication management not only during the hospital stay but especially at discharge for treatments to be continued at home (St. Peter et al., 2013; Alicic et al., 2016; Al Raiisi et al., 2019; Molnar et al., 2020). Moreover, the intervention of pharmacists has a positive impact not only outcomes, but also on the humanistic and economic outcomes of patients affected by CKD (Salgado et al., 2012).

### Limits and Strengths

The analysis of real-world data in older patients with a high grade of complexity, who are constantly excluded from premarketing studies, is a major strength of this analysis. Furthermore, the multicenter design of the REPOSI register and the large number of internal medicine and geriatric wards involved make the study representative of the Italian real-world scenario. Moreover, this could be considered as the first overview of factors associated with contraindicated drug prescriptions in a large cohort of older adults affected by CKD and hospitalized in internal medicine and geriatric settings, performed in a long-time study period, and focused on differences in drug use between admission and discharge. Other studies based on nephrology departments, general practice setting or a single drug class are available but the role of a closer collaboration between the clinical pharmacologist with the general practitioner and the hospital physician in the management of hospitalized older patients affected by CKD was not fully defined (Laville et al., 2018; Okoro and Farate, 2019; Barbieri et al., 2020). Nevertheless, this study has some limitations. First, the lack of completed data, especially related to albuminuria laboratory values, did not detect all patients affected by CKD. Second, the inability to accurately know the real beginning of CKD, as well as the possible, but not probable, misclassification of CKD with acute kidney injury diagnosis, cannot be excluded.

## Conclusion

The present study highlights an overall increase in therapeutic appropriateness in hospitalized older patients affected by CKD, despite a little percentage of therapeutic inappropriateness also occurred at discharge. For this reason, data could be further improved by paying greater attention to possible therapeutic alternatives. Therefore, the need for a closer collaboration between the general practitioner, the hospital physician and the clinical pharmacologist must be consolidated with the feasible aims of therapeutic appropriateness.

## Data Availability

The dataset generated for this study will not be made publicly available. Further inquires can be directed to the author SC, salvatore.corrao@unipa.it.
