# Supplementary material for: Kidney Disease Management in the Hospital Setting: A Focus on Inappropriate Drug Prescriptions in Older Patients
Source: Front Pharmacol. 2021 Oct 8;12:749711. doi: 10.3389/fphar.2021.749711 (PMC8531549; doi:10.3389/fphar.2021.749711)
Supplement: Supplementary file 1 [file Table1.docx]

**Table S1** List of contraindicated drugs in patients with chronic kidney disease (CKD) on the basis of the Summary of Product Characteristics (SmPC).

| ***Drug class*** | ***Active substance (ATC)*** | ***Notes*** |
| --- | --- | --- |
| ***Agents acting on the renin-angiotensin system*** | benazepril (C09AA07) | eGFR <60 ml/min/1.73m^2^ in combination with aliskiren |
|  | captopril (C09AA01) | eGFR <60 ml/min/1.73m^2^ in combination with aliskiren |
|  | cilazapril (C09AA08) | eGFR <60 ml/min/1.73m^2^ in combination with aliskiren |
|  | enalapril (C09AA02) | eGFR <60 ml/min/1.73m^2^ in combination with aliskiren |
|  | irbesartan (C09CA04) | eGFR <60 ml/min/1.73m^2^ in combination with aliskiren |
|  | lisinopril (C09AA03) | eGFR <60 ml/min/1.73m^2^ in combination with aliskiren |
|  | moexipril (C09AA13) | eGFR <60 ml/min/1.73m^2^ in combination with aliskiren |
|  | olmesartan (C09CA08) | eGFR <60 ml/min/1.73m^2^ in combination with aliskiren |
|  | ramipril (C09AA05) | eGFR <60 ml/min/1.73m^2^ in combination with aliskiren |
|  | quinapril (C09AA06) | eGFR <60 ml/min/1.73m^2^ in combination with aliskiren |
|  | telmisartan (C09CA07) | eGFR <60 ml/min/1.73m^2^ in combination with aliskiren |
|  | trandolapril (C09AA10) | eGFR <60 ml/min/1.73m^2^ in combination with aliskiren |
|  | zofenopril (C09AA15) | eGFR <20 ml/min/1.73m^2^ |
| ***Analgesics*** | acetylsalicylic acid (N02BA01) | eGFR <30 ml/min/1.73m^2^ or with one of the following diagnosis (570-573, 070, 072.71, 428, 416.9) or in combination with methotrexate |
|  | rizatriptan (N02C C04) | eGFR <30 ml/min/1.73m^2^ |
|  | zolmitriptan (N02CC03) | eGFR <15 ml/min/1.73m^2^ |
| ***Antacids*** | aluminium compounds (A02AB*) | eGFR <30 ml/min/1.73m^2^ |
|  | calcium compounds (A02AC*) | eGFR <30 ml/min/1.73m^2^ |
|  | combinations of aluminium, calcium, magnesium compounds (A02AD*) | eGFR <30 ml/min/1.73m^2^ |
|  | magnesium compounds (A02AA*) | eGFR <30 ml/min/1.73m^2^ |
| ***Antibacterials for systemic use*** | nitrofurantoin (J01XE01) | eGFR <40 ml/min/1.73m^2^ |
|  | sulfametoxazolo + trimethoprim (J01EA01) | eGFR <30 ml/min/1.73m^2^ or with one of the following diagnosis (570-573, 070, 072.71, 282.2) |
| ***Antigout preparations*** | colchicine (M04AC01) | eGFR <30 ml/min/1.73m^2^ |
| ***Antihistamines for systemic use*** | cetirizine (R06AE07) | eGFR <10 ml/min/1.73m^2^ |
|  | levocetirizine (R06AE09) | eGFR <10 ml/min/1.73m^2^ |
| ***Antiinflammatory and antirheumatic products*** | aceclofenac (M01AB16) | eGFR <30 ml/min/1.73m^2^ or with one of the following diagnosis (570-573, 070, 072.71, 428, 416.9, 400-414, 440 - 440.30, 440.9, 430-434, 435.8-435.9, 436-437) |
|  | celecoxib (M01AH01) | eGFR < 30 ml/min/1.73m^2^ or with one of the following diagnosis (570-573, 070, 072.71, 428, 416.9, 400-414, 440 - 440.30, 440.9, 430-434, 435.8-435.9, 436-437) |
|  | dexketoprofen (M01AE17) | eGFR <60 ml/min/1.73m^2^ or with one of the following diagnosis (570-573, 070, 072.71, 428, 416.9) |
|  | dexibuprofen (M01AE14) | eGFR <30 ml/min/1.73m^2^ or with one of the following diagnosis (570-573, 070, 072.71, 428, 416.9) |
|  | diclofenac (M01AB05) | eGFR <30 ml/min/1.73m^2^ or with one of the following diagnosis (570-573, 070, 072.71, 428, 416.9, 400-414, 440 - 440.30, 440.9, 430-434, 435.8-435.9, 436-437);  in combination with misoprostol eGFR <30 ml/min/1.73m^2^ and with the diagnosis of at least one of the following diseases (070, 072.71, 130.5, 573.1, 573.2, 573.3, 130.5, 570, 571.1-571.2-571.3-571.4, 571.5,428, 416.9) |
|  | etoricoxib (M01AH05) | eGFR <30 ml/min/1.73m^2^ or with one of the following diagnosis (570-573, 070, 072.71, 428, 416.9, 400-414, 440 - 440.30, 440.9, 430-434, 435.8-435.9, 436-437) |
|  | ibuprofen (M01AE01) | eGFR <30 ml/min/1.73m^2^ or with one of the following diagnosis (570-573, 070, 072.71, 428, 416.9) |
|  | ketoprofen (M01AE03) | eGFR <30 ml/min/1.73m^2^ or with one of the following diagnosis (428, 416.9) |
|  | ketorolac (M01AB15) | eGFR <60 ml/min/1.73m^2^ or with one of the following diagnosis (070, 072.71, 130.5, 573.1, 573.2, 573.3, 130.5, 570, 571.1-571.2-571.3-571.4, 571.5,428, 416.9) |
|  | lornoxicam (M01AC05) | eGFR <30 ml/min/1.73m^2^ or with one of the following diagnosis (570-573, 070, 072.71, 428, 416.9) |
|  | meloxicam (M01AC06) | eGFR <30 ml/min/1.73m^2^ or with one of the following diagnosis (570-573, 070, 072.71, 428, 416.9) |
|  | naproxen (M01AE02; M01AE52; M01AE56) | eGFR <30 ml/min/1.73m^2^ or with one of the following diagnosis (428, 416.9);  in combination with esomeprazole eGFR <30 ml/min/1.73m^2^ and with the diagnosis of at least one of the following diseases (570-573, 070, 072.71, 428, 416.9) |
|  | niflumic acid (M01AX02) | eGFR <30 ml/min/1.73m^2^ or with one of the following diagnosis (570-573, 070, 072.71, 428, 416.9) |
|  | nimesulide (M01AX17) | eGFR <30 ml/min/1.73m^2^ or with one of the following diagnosis (570-573, 070, 072.71, 428, 416.9) |
|  | piroxicam (M01AC01) | eGFR <30 ml/min/1.73m^2^ or with one of the following diagnosis (570-573, 070, 072.71, 428, 416.9, 400-414, 440 - 440.30, 440.9, 430-434, 435.8-435.9, 436-437) |
|  | tenoxicam (M01AC02) | eGFR < 30 ml/min/1.73m^2^ |
|  | tiaprofenic acid (M01AE11) | eGFR < 30 ml/min/1.73m^2^ or with one of the following diagnosis (570-573, 070, 072.71) |
| ***Antineoplastic agents*** | cisplatin (L01XA01) | eGFR <60 ml/min/1.73m^2^ |
|  | methotrexate (L04AX03) | eGFR <30 ml/min/1.73m^2^ or with one of the following diagnosis (570-573, 070, 072.71) |
| ***Anti-Parkinson drugs*** | ropinirole (N04BC04) | eGFR <30 ml/min/1.73m^2^ |
| ***Antithrombotic agents*** | acetylsalicylic acid (B01AC06) | eGFR <30 ml/min/1.73m^2^ or with one of the following diagnosis (570-573, 070, 072.71, 428, 416.9) or in combination with methotrexate |
|  | dabigatran (B01AE07) | eGFR <30 ml/min/1.73m^2^ |
|  | fondaparinux (B01AX05) | eGFR <20 ml/min/1.73m^2^ |
|  | nadroparin (B01AB06) | eGFR<30 ml/min/1.73m^2^ or with the diagnosis of 453.40 |
| ***Beta blocking agents*** | nebivolol (C07AB12) | eGFR < 30 ml/min/1.73m^2^ |
|  | sotalol (C07AA07) | eGFR < 10 ml/min/1.73m^2^ |
| ***Cardiac therapy*** | trimetazidine (C01EB15) | eGFR <30 ml/min/1.73m^2^ |
| ***Calcium channel blockers*** | lercanidipine (C08CA13) | eGFR < 30 ml/min/1.73m^2^ |
|  | manidipine (C08CA11) | eGFR < 10 ml/min/1.73m^2^ |
| ***Diuretics*** | canrenone (C03DA03) | eGFR <30 ml/min/1.73m^2^ (in combination with benazepril eGFR <60 ml/min/1.73m^2^) |
|  | eplerenone (C03DA04) | eGFR <30 ml/min/1.73m^2^ |
|  | hydrochlorothiazide (C03AA03) | eGFR <30 ml/min/1.73m^2^ or with one of the following diagnosis (570-573, 070, 072.71, 275.42) |
|  | indapamide (C03BA11) | eGFR <30 ml/min/1.73m^2^ |
|  | piretanide (C03CA03) | eGFR <60 ml/min/1.73m^2^ |
|  | potassium canrenoate (C03DA02) | eGFR <30 ml/min/1.73m^2^ |
|  | spironolactone (C03DA01) | eGFR <30 ml/min/1.73m^2^ |
| ***Drugs for treatment of bone diseases*** | alendronic acid (M05BB03) | eGFR <30 ml/min/1.73m^2^ |
|  | risedronic acid (M05BA07) | eGFR <35 ml/min/1.73m^2^ |
|  | zoledronic acid (M05BA08) | eGFR <35 ml/min/1.73m^2^ |
| ***Drugs used in diabetes*** | acarbose (A10BF01) | eGFR <25 ml/min/1.73m^2^ |
|  | glibenclamide (A10BB01) | eGFR <30 ml/min/1.73m^2^ |
|  | glicazide (A10BB07) | eGFR <30 ml/min/1.73m^2^ |
|  | glimepiride (A10BB12) | eGFR <30 ml/min/1.73m^2^ |
|  | glipizide (A10BB07) | eGFR <30 ml/min/1.73m^2^ |
|  | metformin (A10BA02) | eGFR <30 ml/min/1.73m^2^ |
| ***Immunostimulants*** | interferon alfa-2b (L03AB05) | eGFR <30 ml/min/1.73m^2^ |
| ***Immunosuppressants*** | leflunomide (L04AA13) | eGFR <60 ml/min/1.73m^2^ |
| ***Lipid modifying agents*** | bezafibrate (C10AB02) | eGFR <60 ml/min/1.73m^2^ |
|  | fenofibrate (C10AB05) | eGFR <30 ml/min/1.73m^2^ |
|  | gemfibrozil (C10AB04) | eGFR <30 ml/min/1.73m^2^ |
|  | rosuvastatin (C10AA07) | eGFR <30 ml/min/1.73m^2^ |
| ***Mineral supplements*** | calcium compounds (A12A*) | eGFR <30 ml/min/1.73m^2^ |
|  | magnesium compounds (A12CC*) | eGFR <30 ml/min/1.73m^2^ |
|  | potassium compounds (A12B*) | eGFR <30 ml/min/1.73m^2^ |
| ***Ophthalmologicals*** | brinzolamide (S01EC04) | eGFR <30 ml/min/1.73m^2^ |
|  | dorzolamide (S01EC03) | eGFR <30 ml/min/1.73m^2^ |
| ***Pituitary and hypothalamic hormones and analogues*** | desmopressin (H01BA02) | eGFR <30 ml/min/1.73m^2^ |
| ***Psychoanaleptics*** | duloxetine (N06AX21) | eGFR <30 ml/min/1.73m^2^ |
|  | galantamine (N06DA04) | eGFR <9 ml/min/1.73m^2^ |
| ***Psycholeptics*** | clozapine (N05AH02) | eGFR <30 ml/min/1.73m^2^ |
|  | lithium (N05AN01) | eGFR <60 ml/min/1.73m^2^ |
| ***Sex hormones and modulators of the genital system*** | danazol (G03XA01) | eGFR <30 ml/min/1.73m^2^ |
|  | raloxifene (G03XC01) | eGFR <30 ml/min/1.73m^2^ |
| ***Urologicals*** | alfuzosin (G04CA01) | eGFR <30 ml/min/1.73m^2^ |
|  | vardenafil (G04BE09) | eGFR <30 ml/min/1.73m^2^ |

*eGFR,* estimated glomerular filtration rate.

All diagnoses were codified according to the International Classification of Diseases code, 9th revision (ICD9).

All drugs were codified according to the Anatomical, Therapeutic and Chemical Classification System (ATC)
